# Platinum clusters with precise numbers of atoms for preparative-scale catalysis

**DOI:** 10.1038/s41467-017-00800-4

**Published:** 2017-09-25

**Authors:** Takane Imaoka, Yuki Akanuma, Naoki Haruta, Shogo Tsuchiya, Kentaro Ishihara, Takeshi Okayasu, Wang-Jae Chun, Masaki Takahashi, Kimihisa Yamamoto

**Affiliations:** 1 Laboratory for Chemistry and Life Science, Institute of Innovative Research, Tokyo Institute of Technology, Yokohama, 226-8503 Japan; 2 ERATO-JST, Saitama, Kawaguchi 332-0012 Japan; 3 PRESTO-JST, Saitama, Kawaguchi 332-0012 Japan; 4 Graduate School of Arts and Sciences, International Christian University, Tokyo, 181-8585 Japan; 5 Department of Applied Chemistry, Faculty of Engineering, Yamanashi University, Kofu, 400-8501 Japan

## Abstract

Subnanometer noble metal clusters have enormous potential, mainly for catalytic applications. Because a difference of only one atom may cause significant changes in their reactivity, a preparation method with atomic-level precision is essential. Although such a precision with enough scalability has been achieved by gas-phase synthesis, large-scale preparation is still at the frontier, hampering practical applications. We now show the atom-precise and fully scalable synthesis of platinum clusters on a milligram scale from tiara-like platinum complexes with various ring numbers (*n* = 5–13). Low-temperature calcination of the complexes on a carbon support under hydrogen stream affords monodispersed platinum clusters, whose atomicity is equivalent to that of the precursor complex. One of the clusters (Pt_10_) exhibits high catalytic activity in the hydrogenation of styrene compared to that of the other clusters. This method opens an avenue for the application of these clusters to preparative-scale catalysis.

## Abstract (web summary)

The catalytic activity of a noble metal nanocluster is tied to its atomicity. Here, the authors report an atom-precise, fully scalable synthesis of platinum clusters from molecular ring precursors, and show that a variation of only one atom can dramatically change a cluster’s reactivity.

## Introduction

Noble metal nanoparticles are of particular importance in terms of emerging technologies requiring highly active catalysts for fuel cells^1^, gas reforming^2^, and even fine chemical conversion using tandem flow reactor systems^3^. Although catalytic activities of conventional particles moderately depend on the particle size mainly due to the differing surface-to-volume ratios^4^, evolution of edges^5^, kinks, steps^6^, or higher plane indices^7^ occasionally elicits drastic changes in their properties. From the standpoint of these deliberate defect implementations, extreme miniaturization of the particle is the ultimate approach because subnanometer particles consequently have a specific structure that has no periodicity against crystalline nanoparticles. Such clusters on a subnanometer scale indeed showed distinct properties especially for catalytic applications due to above-mentioned geometric nature or electronically induced superatomic character elucidated by early studies^8–12^. To study the specific activities, a gas-phase synthesis with the mass selection of each cluster has been the only way providing the atomicity control with satisfactory precision and scalability^9, 13–18^. However, this approach is only applicable for analytical-scale catalysis due to the limited throughput of the mass selection.

As one of the few pioneering examples of this scale-up, we recently reported a dendrimer-based fine cluster synthesis method that allowed atomicity control with single-atom precision^19^, and found that the catalytic activity was highly sensitive to the atomicity between 12 and 20^20^. The overall trend indicated that a metastable cluster provided a better catalytic performance^21^. If such a precise large-scale synthesis could be applied to every atomicity with a much easier procedure, the cluster science would allow to access the next practical level, taking full advantage of the systematic implementation of such an atomicity-specific metastable nature.

Platinum is the most important metal element for nanoparticle catalysis. As the precursors of such ultrasmall platinum subnanoparticles, platinum thiolates are currently being investigated because reductive metal-sulfur bond cleavage might lead to the formation of bare metal clusters. The thiolates can form tiara-like cyclic complexes stable enough for isolation^22–27^. In principle, such tiara-like complexes are available with atomicities of 4 and higher^28^. This might allow fully scalable synthesis regardless of the specific stable magic numbers just like the gas-phase synthesis. Although several syntheses from stable ligand-protected “magic number” clusters^29, 30^ or multinuclear complexes^31, 32^ have been attempted, these precursors were not scalable, and the exact definition of the atomicity was never demonstrated. One of the issues is the high metal-to-ligand binding energy, which requires extremely high calcination temperatures leading to the aggregation^10^.

Here we show the scalable synthesis, characterization, and fundamental properties of platinum tiara-like complexes with octanethiol ligands. The synthesis of monodispersed subnanometer platinum clusters and their catalytic activity for preparative-scale reactions are investigated by achieving the non-destructive quantitative conversion from the complexes to the clusters.

## Results

### Synthesis of precursor complexes

Only one example of a platinum thiolate tiara-like complex with a full chemical identification (nuclear magnetic resonance (NMR), mass, and elemental analysis) has been published^33^. Therefore, we began this study with the investigation of the basic reactions between PtCl_4_ and *n*-octanethiol (C_8_H_17_SH). The reaction at room temperature in a chloroform/acetonitrile solution was monitored by positive-ion matrix-assisted laser-desorption-ionization time-of-flight mass spectrometry (MALDI-TOF-MS) using *trans*-2-[3-(4-*tert*-butylphenyl)-2-methyl-2-propenylidene]malononitrile (DCTB) as the matrix^34^. After 3 h, many peaks below 5000 Da associated with mono-cationic species were observed. Some of them could be attributed to the desired [Pt(µ-SC_8_H_17_)_2_]_*n*_^+^ (*n* = 5–9) species, including their isotropic patterns, while each of them was accompanied by a minor species with an additional 216 Da mass. Because this additional mass is equivalent to the sum of C_8_H_17_SH and Cl_2_, these minor species are thought to be linear oligomers, [Pt(µ-SC_8_H_17_)_2_]_*n*_(SC_8_H_17_)_2_Cl_2_, detected as fragment [M-SC_8_H_17_]^+^ ions. Based on this initial result, it is anticipated that platinum thiolates initially undergo a linear chain growth reaction^35^ followed by the entropically favorable formation of smaller cyclic compounds^36^. Based on this idea, the synthesis protocol was optimized in two steps. PtCl_4_ was reacted with four equivalents of *n*-octanethiol (C_8_H_17_SH) in a chlorobenzene/acetonitrile (1/1) mixed solvent at 90 °C in the presence of diisopropylethylamine (DIEA). After 1 h, the reaction mixture was concentrated, and further treated in chlorobenzene with excess C_8_H_17_SH at 125 °C for an additional hour. The crude product containing the tiara-like complexes with various ring sizes [Pt(µ-SC_8_H_17_)_2_]_*n*_ was obtained in an overall 36% yield.

**Fig. 1:**
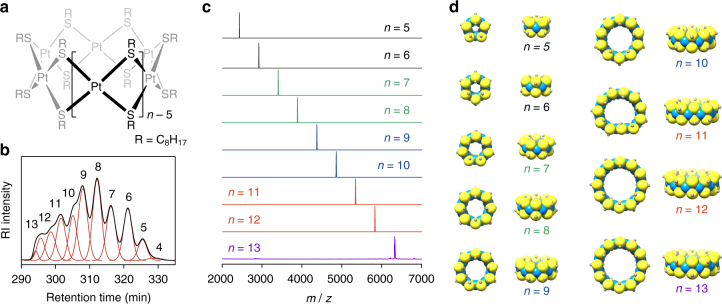
Tiara-like platinum octanethiolate complexes [Pt(µ-SC_8_H_17_)_2_]_*n*_. **a** Chemical structure of the complexes. **b** Chromatogram of the preparative HPLC separation with size-exclusion columns (*SEC*) monitored by a refractive index detector. This chromatogram was recorded at five cycles of the recycling processes. **c** MALDI-TOF-mass spectra of the isolated [Pt(µ-SC_8_H_17_)_2_]_*n*_ (*n* = 5–13) measured with DCTB matrix. **d** Optimized geometric structures at the B3LYP/LanL2DZ level. Platinum, sulphur, and α-carbon atoms are shown as *blue*, *yellow*, and *gray spheres*, respectively. The hydrogen atoms are omitted for clarity

All the tiara-like complexes [Pt(µ-SC_8_H_17_)_2_]_*n*_ (Fig. 1a: *n* = 5–13) were isolated by size-exclusion chromatography. The hexane-soluble part of the product was extracted as a crude mixture, then subjected to preparative size-exclusion chromatography. Finally, pure complexes with different ring sizes could be isolated up to 25 recycling processes by our apparatus (see Methods section). MALDI-TOF-MS of the product mixture before the isolation showed the presence of various complexes [Pt(µ-SC_8_H_17_)_2_]_*n*_. The highest peak intensities were observed at *n* = 8 and 9, whereas even a high molecular weight complex (*n* ~ 30) was found. Remarkably, every complex (5 ≤ *n* ≤ 30) had a molecular weight attributed to the cyclic tiara-like structures. The refractive index chromatogram after several recycling processes exhibited many independent peaks (Fig. 1b), which were successfully characterized by ring size using MALDI-TOF-MS based on their isotope patterns. The isolations were their successful for complexes with ring sizes between *n* = 5 and 13 (Fig. 1c). All the compounds except for [Pt(µ-SC_8_H_17_)_2_]_13_ were fully characterized by ^1^H NMR, ^13^C NMR, and elemental analyses in addition to MALDI-TOF-MS (Supplementary Methods). Single-crystal X-ray structures were successfully obtained for *n* = 6 and 8 (Supplementary Figs. 1, 2; Supplementary Table 1) showing their tiara-like macrocyclic structures. Although crystallographic data were not available for the other rings due to their poor crystallinity, all the given structures were optimized (Fig. 1d) at the B3LYP/LanL2DZ level using Gaussian 09^37^. To reduce the calculation cost, methylthiol (CH_3_SH) was used instead of octanethiol (C_8_H_17_SH) as the ligand.

### Conversion to atom-precise clusters

The thermostability of the complexes was next investigated in order to reveal their potential as precursors for atom-precise platinum clusters. Under a helium atmosphere, it was determined that the coordinating thiolate ligands start being eliminated at 200 °C. A thermogravimetric-differential thermal analysis with mass spectrometry (TG-DTA-MS) indicated that the C–S bonds of the thiolate ligands were cleaved first, followed by the Pt–S bond cleavages (Supplementary Fig. 3). Although these bond cleavages for the palladium thiolates occurred completely stepwise^38^, the present platinum thiolate almost concurrently underwent two cleavages. This result suggested that selective conversion of the tiara-like platinum complexes to the corresponding platinum (metal) or platinum sulfide clusters is in principle difficult. However, the reductive Pt–S bond cleavages for the selective production of zero-valent platinum clusters were possible under a hydrogen gas stream. When a thick [Pt(µ-SC_8_H_17_)_2_]_*n*_ film on a glass substrate was heated at 250 °C for 2 h under a pure H_2_ stream, a metallic film was formed. It was determined to be polycrystalline platinum by an X-ray diffraction (XRD) measurement (Supplementary Fig. 4).

**Fig. 2:**
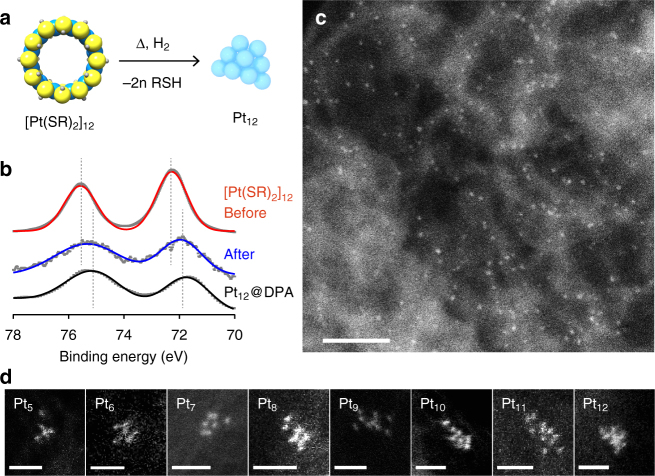
Preparation of monodispersed Pt_12_ clusters from [Pt(µ-SC_8_H_17_)_2_]_12_ complexes. **a** Direct conversion scheme from a complex to a cluster by reduction in a hydrogen atmosphere. **b** XPS of the [Pt(µ-SC_8_H_17_)_2_]_12_ complexes in Pt 4*f*_7/2_ and Pt 4*f*_5/2_ regions before and after calcination at 250 °C under a hydrogen stream. Pt_12_@DPA is the previously synthesized sample prepared with a phenylazomethine dendrimer in ref. ^19^. **c** A dark-field STEM image of the calcined sample supported on carbon (Ketjenblack). Inset shows a high-magnification aberration-corrected STEM image. *Scale bar*, 20 nm. **d** Atomic scale high-magnification STEM images of the monodispersed clusters (Pt_5_–Pt_12_). *Scale bar*, 1 nm

[Pt(µ-SC_8_H_17_)_2_]_*n*_ were supported on carbon (Ketjenblack) in order to preserve the atomicity of the precursor during the reduction (Fig. 2a). After a low-temperature (250 °C) calcination of the supported [Pt(µ-SC_8_H_17_)_2_]_*n*_ for 2 h, X-ray photoelectron spectra (XPS) of the products indicated that a trace amount of sulfur remained on the carbon-supported cluster. Further optimization of the calcination conditions determined that the formation of zero-valent Pt (Pt 4*f*_7/2_: Fig. 2b) and complete removal of the thiolate ligand (S3*p*_3/2_: Supplementary Fig. 5) were possible using a longer reduction time (18 h) with a high H_2_/N_2_ (3%) flow rate. In the case of [Pt(µ-SC_8_H_17_)_2_]_12_, the XPS was almost identical to that for Pt_12_ produced by the dendrimer-reactor method^19^. HAADF-STEM images of the as-synthesized clusters indicated monodispersed Pt clusters supported on the carbon (Fig. 2c), with sizes similar to those previously reported for Pt_12_^39^. The most important detail is that the procedure is universal for other atomicities derived from various complexes [Pt(µ-SC_8_H_17_)_2_]_*n*_ (*n* = 5–12). The sizes of all of the particles in the STEM images were close to that expected based on the structural models (Supplementary Figs. 6, 7).

The histograms of each cluster size are apparently monodisperse accompanying small distributions. It is important to note that these distributions are not only due to the distributions in the cluster atomicity, but also due to the different projected shapes and the inevitable defocus by the strictly limited focus depth. Thus, we suggest that quantitative evaluation based on the size distribution is at the limit at the subnanometer scale. Instead, detailed study on the precision of atomicity control by nucleation of the atom-precise precursor is possible by the observation at the subatomic scale. For example, Ketjenblack as a support loading [Pt(µ-SC_8_H_17_)_2_]_8_ at the weight percentage of 1.0 wt% was investigated by high-resolution HAADF-STEM. Although the atomicity was completely retained before the reduction, the number of observed particles decreased after the reductive calcination. Larger particles containing 16, 24, or more Pt atoms were also observed, suggesting the aggregation during the reduction (Supplementary Fig. 8). Increase of the platinum loading to 1.8 wt% resulted in promotion of the cluster aggregation producing nano-crystalline particles with ca. 2 nm in the diameters (Supplementary Fig. 9). In contrast, the decrease of the loading to 0.4 wt% significantly suppressed this aggregation. Conversely, production of smaller clusters due to the decomposition was hardly observed. In this case, the atomicities of each cluster are almost preserved from the precursor complex (Supplementary Fig. 10). For the statistical analysis of the particle aggregation, the numbers of particles before and after the reductive calcination were counted (Supplementary Fig. 11). Residual ratio (*R*) is defined as *R* = *N*_after_/*N*_before_ where *N*_before_ and *N*_after_ are the numbers of observed particles within a sampling area. If the particles did not aggregate at all, *R* should be 1. Average particle-to-particle distance was derived from the number of Pt–thiolate complexes (before the calcination) in the sampling area of the projected STEM image; therefore, it does not describe the actual distances between the precursors but the distances as seen on the STEM image. Increase of the Pt loading (wt%) on the carbon support (Ketjenblack) decreases the distance. This analysis suggests that the original particle could be preserved if the particle-to-particle distance was longer than ca. 7 nm on the STEM image. This optimized method allowed the selective and atom-precise synthesis of clusters ranging from Pt_5_ to Pt_12_ as shown by the atomic resolution HAADF-STEM images (Fig. 2d). Based on the investigations, the cluster samples for the following structural and catalytic examinations were prepared with a net platinum content of ≤0.4 wt% or less. Details of the sample preparations are described in the Methods section.

Atomic-level electronic and structural information, such as the valence states, coordination numbers, and interatomic distances, was investigated by Pt-4*f* XPS and Pt-*L*_3_ EXAFS spectroscopy of the resulting Pt clusters (*n* = 8). As mentioned above, the as-synthesized clusters were reduced, but the binding energies were higher than that of the bulk platinum metal (70.9 eV). As previously reported, this is considered to result from partial oxygen adsorption to the surface and the final-state effect^40^. The dependence on the size of the cluster is not significant in the range of the atomicity investigated in this study (Supplementary Figs. 12 and 13), but this trend is consistent with the previous mass-selected platinum clusters synthesized by the vacuum-phase method^17^. The EXAFS spectrum of [Pt(µ-SC_8_H_17_)_2_]_8_ calcined under a H_2_/N_2_ stream was different from that for the untreated complex (Supplementary Figs. 14 and 15). A curve-fitting analysis elucidated that the average Pt–Pt distance was 2.71 Å. The very small coordination number (CN = 3.5 ± 2.1) was within the range of the expected values for Pt_8_^41^, supporting the formation of tiny clusters without nonnegligible aggregation (Supplementary Table 2). In addition to the Pt–Pt bonds, consideration of the adjacent light elements (C or O) provided a better curve-fitting result. It should be noted that the contribution of Pt-S shell cannot be excluded from the analysis of EXAFS. Based on the XPS results, i.e., that calcined Pt_8_ indicated significantly low amount of S atoms relative to the thiolate precursors, the possible adjacent atoms would be the carbons on the Ketjenblack. The large coordination number (CN = 6.6) for the relatively long Pt-C (2.17 Å) may suggest a flat structure of Pt_8_ on the graphitic carbon substrate.

### Catalytic activity

The catalytic study of atomically precise clusters is limited to recent pioneering experiments such as the reactions of gaseous molecules with gas-phase clusters beam or soft landed clusters on a flat substrate^9, 13–18^. However, none of the activity, selectivity, and durability under practical conditions were studied. The present method provides a new pathway for the large-scale production of atom-precise clusters. As the demonstration of preparative-scale catalysis, we performed the hydrogenation of styrene and the aerobic oxidation of neat indane.

**Fig. 3:**
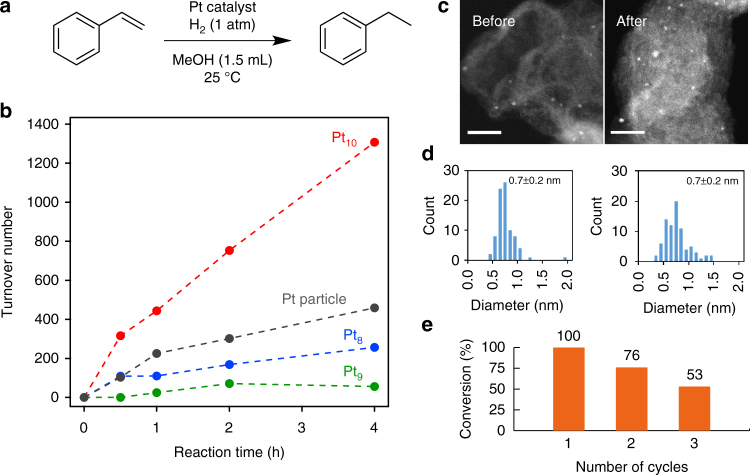
Hydrogenation reaction of styrene catalyzed by platinum clusters. **a** A scheme of the conversion. **b** Turnover numbers of the styrene conversion catalyzed by platinum clusters (Pt_8_, Pt_9_, and Pt_10_) on Ketjenblack (Pt: 0.02 mol%). Pt particle represents the platinum nanoparticles (1.1 ± 0.2 nm) prepared from [Pt(µ-SC_8_H_17_)_2_]_9_ at higher concentration (1.8 wt% Pt) on Ketjenblack. **c** HAADF-STEM images of the Pt_10_ catalyst before and after (2 h) the hydrogenation reaction. *Scale bar*, 10 nm. **d** The particle size histograms from the images on panel **c** and other views. **e** Styrene conversions at the end of the cycles during repeated uses of Pt_10_/KB. The reaction conditions are the same as those in panel **b**, but the amount of catalyst (Pt: 0.1 mol%) is different

The cluster catalysts exhibited characteristic property in the hydrogenation reaction of styrene (Fig. 3a), which has been investigated by the platinum nanoparticles^42, 43^. While no reaction was confirmed by blank Ketjenblack, Pt_8_ cluster supported on Ketjenblack (Pt_8_/KB) as a catalyst clearly promoted the hydrogenation reaction of styrene, suggesting that the platinum clusters act as the catalyst. Ethylbenzene as the product was formed immediately after the initiation of the reaction, and was increased with the reaction time (Fig. 3b). Similar catalytic hydrogenation was also investigated on other platinum clusters (Pt_9_/KB, Pt_10_/KB, and Pt_x_/KB as larger Pt nanoparticles shown in Supplementary Fig. 16) used as the catalyst. As a remarkable point, these clusters showed different catalytic activity despite the only one atom difference of the composition. Among these clusters, Pt_10_/KB exhibited the highest activity.

We can now investigate the characteristics of catalysts not only for the reactivity but also for the new aspects such as degradation of the cluster in the reaction or the possibility of reuse, because it has become possible to obtain clusters with the atomic precision on the preparative scale. Although very low stability of such small clusters is anticipated, the Pt_10_/KB catalyst after the hydrogenation reaction for 2 h observation of the atomic resolution STEM showed unexpectedly retained without obvious aggregation and decomposition (Fig. 3c, d). More careful observation at the atomic scale revealed the existence of single atoms probably arising from the elimination from clusters. However, the frequency was not significant. Based on this characteristic of the present supported cluster catalyst, it is possible to conduct a reuse experiment, in which the product is removed after the reaction, followed by the addition of the substrate to restart (Fig. 3e). The durability of clusters is not excellent at this time, but it would be possible to produce a cluster catalyst with higher durability by changing the cluster composition or improving the interaction with the support.

In contrast, in reactions under oxidative conditions, cluster degradation were more pronounced. Under an oxygen atmosphere, Pt_8_, Pt_9_, or Pt_10_ supported on Ketjenblack were added to freshly distilled indane, and the reaction was carried out at 90 °C. After 6 h, oxidized products including 1-hydroperoxy-indane, 1-indanol, and 1-indanone were observed by ^1^H NMR. In this oxidation reaction, the dependence on the cluster atomicity is hardly observed, and an induction period of several hours was observed until the production of the product. The atomic-level investigation revealed that the atomicity of each cluster changes due to the formation of many single atoms (Supplementary Fig. 17). Therefore, under this oxidative condition, it is strongly indicated that the true active species is not the original cluster. The atomicity of the clusters after the hydrogenation reaction at room temperature was rather preserved relative to the oxidation condition at 90 °C.

## Discussion

In general, catalytic properties of metal nanoparticles are discussed based on surface-to-volume ratio, surface structure^44^, or *d*-band center^45^ as shown in the studies of many metal nanoparticles^4^. However, these discussions cannot be applied to the present cluster series with such low atomicites. For example, the Pt_8_, Pt_9_, and Pt_10_ cluster already exposes all of their atoms on the surface. The surface of these clusters are not attributed to any specific surface index because the clusters do not have lattice structures. In addition, difference in the electronic state of the clusters are not significant as shown by the valence states of the clusters characterized by the XPS measurements (Supplementary Fig. 13). Based on this idea, it is considered that the difference in the activity cannot be simply explained by the conventional discussions.

**Fig. 4:**
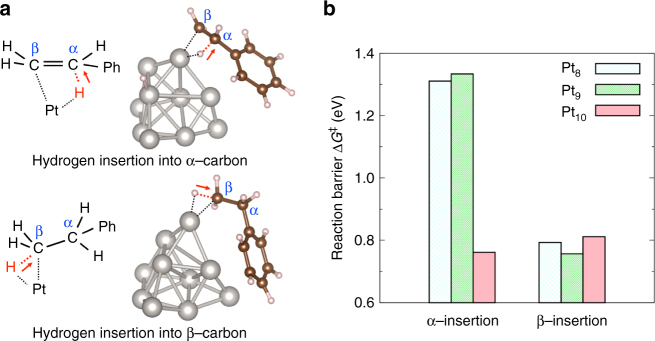
Styrene hydrogenation and its reaction barriers under the existence of platinum catalysts. **a** Hydrogen insertion processes into α-carbon and β-carbon, and the corresponding transition states with Pt_10_ catalyst. **b** Calculated reaction barriers under the existence of Pt_8_, Pt_9_, or Pt_10_ catalyst. Density functional theory calculations were performed at the B3LYP/LanL2DZ and 6–31 G (d, p) level of theory

In order to elucidate the specific enhancement of styrene hydrogenation by Pt_10_, density functional theory calculations were performed with the B3LYP functional, LanL2DZ basis set for platinum atoms, and 6–31 G (d, p) basis set for hydrogen and carbon atoms, using Gaussian 09^37^. Styrene hydrogenation consists of two processes: hydrogen insertions into α-carbon and β-carbon, as shown in Fig. 4a. Accordingly, reaction barriers of these processes were calculated under the existence of Pt_8_, Pt_9_, or Pt_10_ catalyst. Obtained reaction barriers are shown in Fig. 4b. The corresponding transition states with Pt_10_ are illustrated in Fig. 4a, and those with Pt_8_ or Pt_9_ are available in Supplementary Fig. 18. Figure 4b shows that the α-insertion requires high activation energy with Pt_8_ or Pt_9_, and thus is the rate-determining step. On the other hand, Pt_10_ catalyst was found to lower the reaction barrier of the α-insertion considerably. This difference of the energy barriers might be attributed to both electronic and geometrical factors. According to the recent theoretical studies^48, 49^, Pt_8_ and Pt_9_ favor (quasi-)two-dimensional structures, whereas Pt_10_ prefers a rigid pyramidal one. Our calculation shows that platinum atoms at the vertices of the pyramidal Pt_10_ are very electron-rich, which can activate a carbon double bond effectively. In contrast, Pt_8_ and Pt_9_ do not have such electron-rich sites. The calculated charge distributions are shown in Supplementary Fig. 19. Furthermore, non-rigid structures of Pt_8_ and Pt_9_ might interact with phenyl group of styrene strongly, which would impose unfavorable geometrical constraints on the reactant during the reaction. This is one of the plausible reasons why Pt_10_ catalyst facilitates styrene hydrogenation specifically. According to our calculations, atomicity of platinum catalyst does not affect the reaction barrier of the insertion into β-carbon, which is sufficiently low regardless of atomicity.

Although catalytic properties of relatively larger clusters have been discussed based on theoretical calculations^46, 47^, it is, in principle, difficult to uniquely determine its structure and activity in a smaller cluster composed of 1–10s of atoms because the structure dynamically changes as observed in STEM. Our calculation result represents only one of the most probable reaction path of the catalysis. Strictly speaking, it is necessary to consider all of the multiple metastable structures and all the corresponding reaction pathways for the quantitative evaluation.

Recent theoretical calculations explain that Pt_10_ is a magic cluster having a more stable and less fluxional structure than other clusters, and there are fewer isomers^48,^ ^49^. Some of the previous gas-phase investigations suggested that the Pt_10_ cluster is stable but unreactive^50–52^. Apparently, these claims seem to be inconsistent with our experimental and theoretical results. However, the atomicity dependence should be much different by the substrate molecules. In addition, the present hydrogenation reactions were performed in hours order. Thus, it can also be considered that the resistance to cluster dissociation and aggregation was also essential for the high turnover numbers as expected from the STEM observation after the reaction. In the case of such a very small cluster, it might also be necessary to consider the differences in the interaction with the carbon support, which should contain a lot of oxygen-containing groups (hydroxyl or ketone). Actually, we have encountered unexpected deactivation of the catalysis as described in the Methods. For a complete understanding of the catalyst system by such very small clusters, further investigation on the stability and the support effect is the next important subject.

In summary, we successfully isolated various tiara-like platinum thiolate complexes, whose atomicity ranged between 5 and 13. A study on the thermal chemical conversion revealed that the tiara-like platinum thiolate complexes were suitable precursors for the synthesis of monodispersed zero-valent platinum clusters with a specific atomicity (*n* = 5–12), which had been inaccessible by previous chemical methods. Especially, the Pt_10_ cluster prepared from the tiara-like complex exhibited an exceptionally high catalytic activity for the hydrogenation reaction of styrene relative to the other clusters (Pt_8_, Pt_9_, or larger one). To the best of our knowledge, this is the first example demonstrating a preparative-scale reaction using atom-precise clusters with a single-digit atomicity. Because a similar procedure is generally possible for the synthesis of nickel and palladium clusters, this method is expected to be useful for producing high-performance catalysts.

## Methods

### Chemicals

PtCl_4_ was purchased from Furuya Metal Co., Ltd. *N,N*-diisopropylethylamine and *n*-octanethiol were purchased from Tokyo Chemical Industry Co. Ltd. Styrene was purchased from Aldrich. All the other chemicals and solvents were purchased from Kanto Chemical Co., Inc. These chemicals were used as received unless otherwise specified.

### Synthesis of platinum thiolate complexes

PtCl_4_ (303 mg, 0.90 mmol) was dissolved in a mixed solvent (monochlorobenzene/acetonitrile = 1/1, 300 mL), and the solution was heated at 90 °C for 1 h in the presence of *N,N*-diisopropylethylamine (69 mmol) and *n*-octanethiol (3.6 mmol). The resulting solution was evaporated to dryness. The crude product was again dissolved in monochlorobenzene (10 mL) containing *n*-octanethiol (9.0 mmol), and reacted at 200 °C for an additional 1 h. During the reaction, the solution color was changed from red to pale yellow. After the reaction, the solution was centrifuged to remove the insoluble part as a precipitate. The soluble part includes tiara-like complexes with various ring numbers as a mixture.

### Isolation of the complex

A preparative recycling HPLC (Japan Analytical Industry: LC908) was used for the purifications using chloroform as the eluent. Two tandemly arrayed size-exclusion columns (Japan Analytical Industry: JAIGEL-2H and JAIGEL-2.5H) were used. After several recycling processes, each fraction was isolated. During the recycling process, larger oligomers were collected first. After the additional recycling processes, each tiara-like complex was collected in order. The collection was completed up to 25 cycles. Each fraction was checked by MALDI-TOF-mass measurement, and then the product was characterized by ^1^H, ^13^C NMR, and elemental analysis (Supplementary Fig. 20; Supplementary Methods).

### Typical procedure for the synthesis of Pt_*n*_ clusters

Ketjenblack, KB (30 mg) was dispersed in hexane (200 mL) by ultrasonic dispersion. Hexane solution of [Pt(C_8_H_17_S)_2_]_*n*_ (5 mL) was added dropwise to the suspension of KB while stirring (500 rpm). After stirring for 15 min, the suspension was filtered under suction through a Millipore filter (0.2 μm pore diameter). The collected fine powder of [Pt(C_8_H_17_S)_2_]_*n*_ supported on KB was treated under hydrogen gas stream (3% H_2_/N_2_, 3 L min^−1^) at 250 °C in a tube furnace (KOYO Thermosystem Inc., KTF040N1-AS) for 18 h. This conversion process from the complexes to the metal clusters (Pt_*n*_/KB) is quantitative.

For the synthesis of monodisperse clusters, weight percentages of platinum content were set to be 0.2 and 0.4 wt% for Ketjenblack, respectively, for Pt_*n*≤6_ and Pt_*n*≥7_. In the case of reference catalyst as the larger platinum nanoparticles, [Pt(C_8_H_17_S)_2_]_9_ supported on KB at 1.8 wt% (Pt atom weight percentage) was used as the precursor.

### Preparation of the bulk Pt film for XRD analysis

A small portion (ca. 0.1 mL) of a high-concentration solution (ca. 0.1 mol L^−1^) of the platinum complex mixture (containing various ring numbers) in chloroform was dropped onto a quartz-glass substrate (1 cm^2^), and was stored in air to give yellowish transparent film until the solvent was evaporated. The thick transparent film was changed to silver metallic film after a process under hydrogen gas stream at 250 °C for 1.5 h.

### Catalytic hydrogenation of styrene for the kinetic study

Hydrogenation of styrene was conducted in a test tube, which was joined to a three-way stopcock, which was connected to vacuum and an argon or hydrogen balloon. Styrene was distilled prior to use. Pt clusters supported on carbon (0.04 μmol Pt) and 1.5 mL solution of the styrene in methanol (0.13 mol L^−1^) was added to the test tube. The reaction vessel was flushed three times each with argon and then with hydrogen gas. The hydrogenation reactions were carried out under pure hydrogen (1 atm) at 25 °C with magnetic stirring (400 rpm). The reaction mixture was analyzed by gas chromatography.

In the evaluation of hydrogenation catalytic activity, it is common to activate the catalyst by hydrogen reduction treatment. However, our examinations suggested that the apparent catalytic activity is significantly decreased by the hydrogen reduction treatment of the catalyst just before the activity test. In addition, it was found that the catalyst turnover was also affected by the distillation of styrene. It should be noted that the pretreatment of the catalyst, the support effect, or the influence of impurities in the reaction mixture are not fully understood. Therefore, the experiments should be conducted with care so that the history of each catalyst was the same. Nonetheless, special attention is needed on the discussion of absolute values of the catalytic activity.

### Catalytic hydrogenation of styrene for the recycling and stability test

In a test tube joined to a three-way stopcock, which was connected to a hydrogen balloon, 4.9 mg of Pt_10_ supported on Ketjenblack (0.1 μmol Pt) was reacted with 1.5 mL solution of styrene in methanol (0.07 mol L^−1^). The hydrogenation reaction was carried out for 2 h under pure hydrogen (1 atm) at 25 °C with magnetic stirring (400 rpm). After the reaction, the reaction mixture was dried under vacuum to remove methanol, styrene, and ethylbenzene. A small part of the sample was used for the STEM observation. The catalyst was further washed with methanol and then dried under vacuum. The recovered catalyst was used in the subsequent reaction. Three cycles of the catalysis were investigated to examine the reusability of the catalyst.

### Calculations of styrene hydrogenation

Geometry optimizations and vibrational analyses were performed for intermediates and transition states of styrene + H_2_ with Pt_8_, Pt_9_, or Pt_10_ catalyst, using the B3LYP functional, LanL2DZ basis set for platinum atoms, and 6–31 G (d, p) basis set for hydrogen and carbon atoms. The most stable structures of platinum clusters reported previously^48, 49^ were employed as initial geometries in these calculations. The obtained intermediate structures have no imaginary frequency. In addition, the transition states have only one imaginary frequency. All the calculations were carried out with the Gaussian 09 program package^37^.

### Data availability

The X-ray crystallographic coordinates for structures reported in this article have been deposited at the Cambridge Crystallographic Data Centre (CCDC), under deposition nos. CCDC 1563559 and 1563560. These data can be obtained free of charge (http://www.ccdc.cam.ac.uk/data_request/cif). All other reported data are available from the corresponding authors on reasonable request.

## Electronic supplementary material

Supplementary information
