# Supplementary material for: Platinum clusters with precise numbers of atoms for preparative-scale catalysis
Source: Nat Commun. 2017 Sep 25;8:688. doi: 10.1038/s41467-017-00800-4 (PMC5613004; doi:10.1038/s41467-017-00800-4)
Supplement: Supplementary file 1 — Supplementary information [file 41467_2017_800_MOESM1_ESM.pdf]

Supplementary Figures and Tables

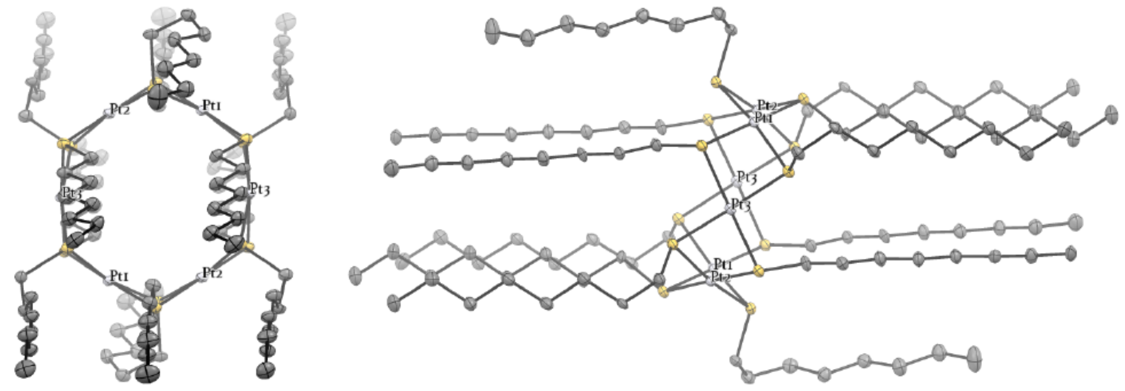

Supplementary Figure 1. Molecular structure of **$[Pt(C_8H_{17}S)_2]_6$**. The hydrogen atoms were omitted for clarity.

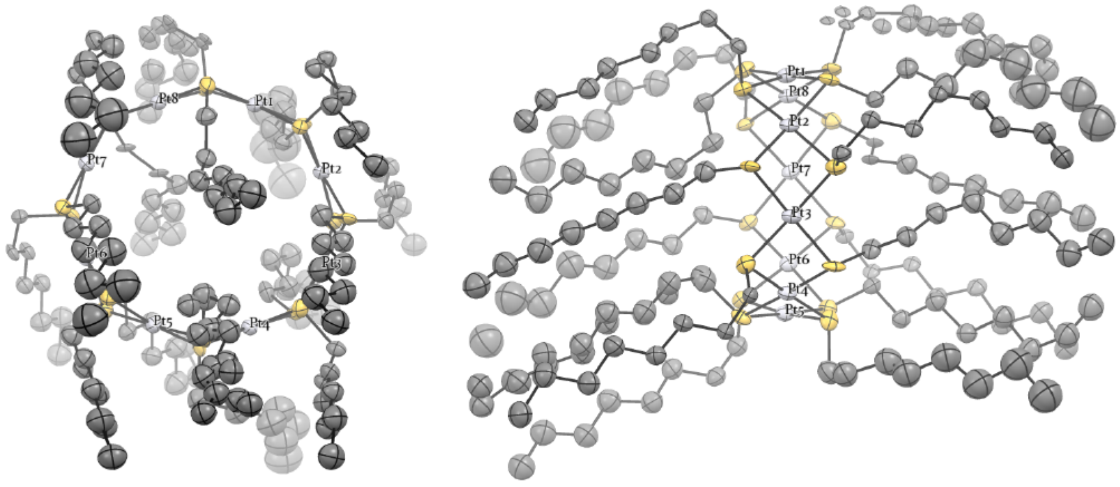

Supplementary Figure 2. Molecular structure of **$[Pt(C_8H_{17}S)_2]_8$**. The hydrogen atoms were omitted for clarity.

Supplementary Table 1. Crystal data and structure refinement for **$[Pt(C_8H_{17}S)_2]_6$** and **$[Pt(C_8H_{17}S)_2]_8$**.

	**$[Pt(C_8H_{17}S)_2]_6$**	**$[Pt(C_8H_{17}S)_2]_8$**
Formula	$C_{96}H_{204}Pt_6S_{12}$	$C_{128}H_{272}Pt_8S_{16}$
Formula weight	2913.84	3885.24
Crystal system	*Triclinic*	*Monoclinic*
Space group	*P*-1 (No.2)	$P2_1$/n (No.14)
a /Å	13.3154(10)	13.95550(6)
b /Å	14.2727(11)	44.9780(2)
c /Å	16.4842(13)	25.19960(10)
α / °	102.9590(10)	90
β / °	102.6240(10)	95.963(4)
γ / °	101.2380(10)	90
V / Å^3	2879.53	15731.96(16)
Z	1	4
F_{000}	1440.00	7680.00
D_{calc} / g cm^{-3}	1.68	1.64
μ(Mo$K\alpha$) / mm^{-1}	7.516	7.308
Temperature / K	90	93
θ range for data collection	1.318° - 29.139°	1.664° - 25.430°
Reflections collected	13406	37683
Independent refluction	11714	18816
Final *R* indices (I > 2δ)	$R_1 = 0.0285$, $wR_2 = 0.0717$	$R_1 = 0.0768$, $wR_2 = 0.2150$
Goodness-of-fit on F^2	1.009	1.002

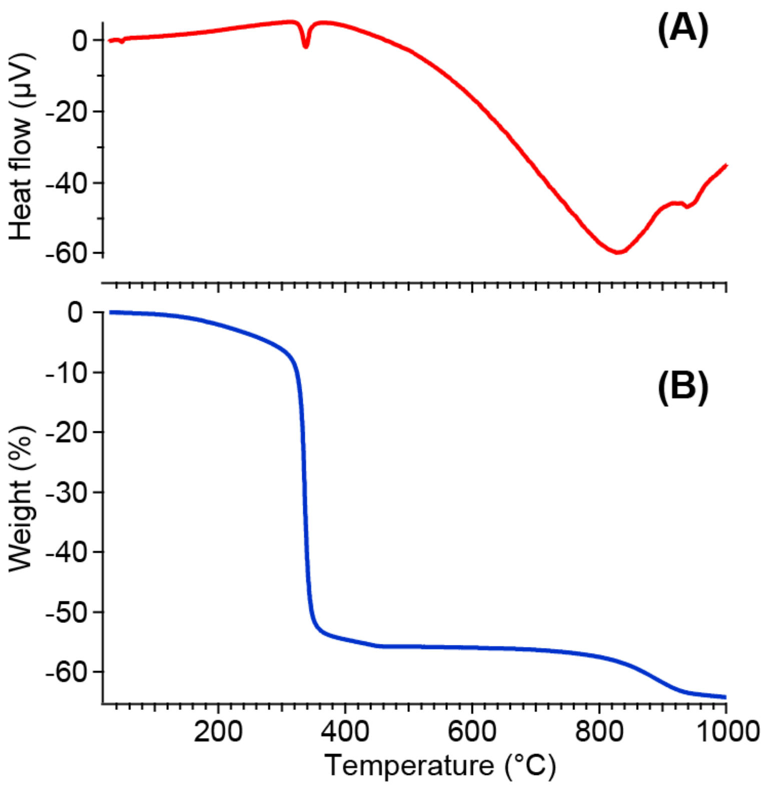

Supplementary Figure 3. (A) Differential thermal analysis (DTA) and (B) thermogravimetric analysis data of **$[Pt(C_8H_{17}S)_2]_6$** measured under a helium atmosphere. Heating rate: 10°C min^{-1}, helium flow rate: 300 ml min^{-1}.

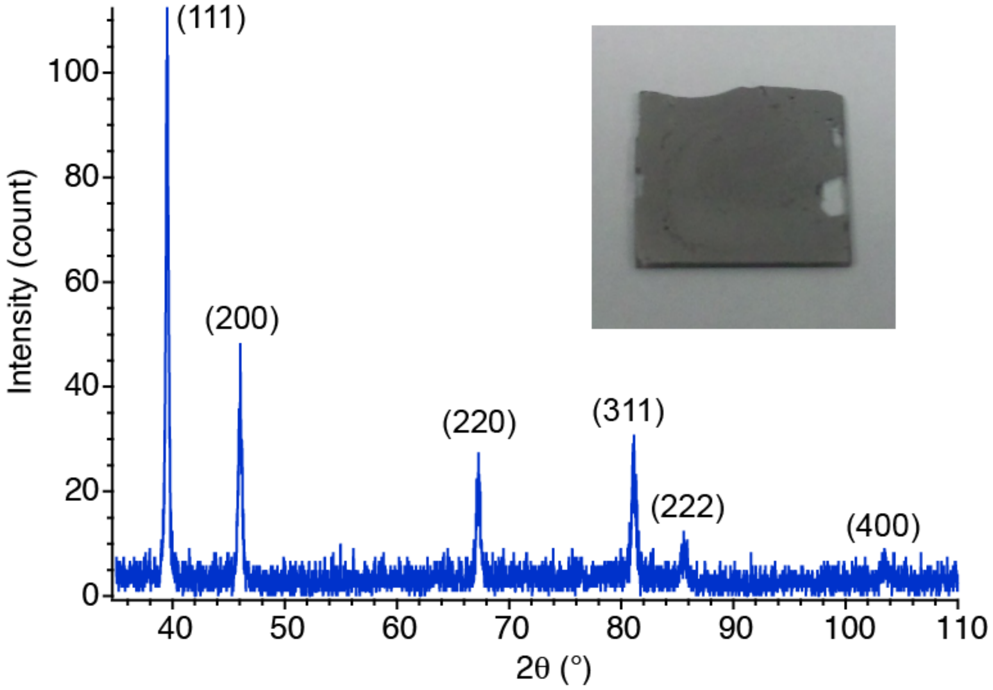

Supplementary Figure 4. Powder X-ray diffraction (XRD) of a thick **$[Pt(C_8H_{17}S)_2]_n$** (mixture of different *n* products) film after a treatment under a hydrogen atmosphere at 250°C. Inset picture is a photograph of the as-prepared product.

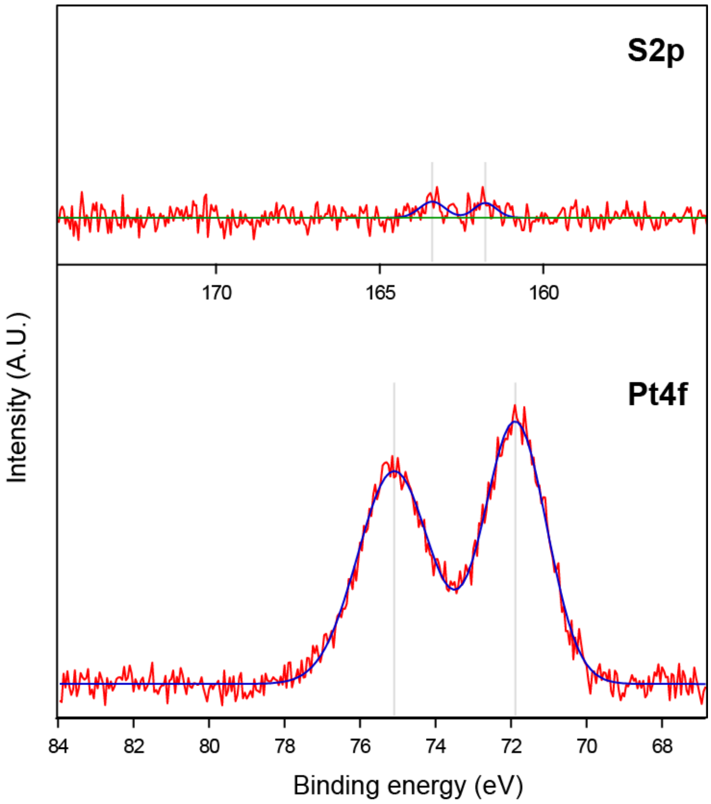

Supplementary Figure 5. X-ray photoelectron spectra (S 2*p* and Pt *4f*) of $\mathbf{Pt_8}$ after the calcination under a hydrogen (3%) / nitrogen (97%) atmosphere at 250°C for 16 hours. Only a trace amount of sulfur was remained after the calcination.

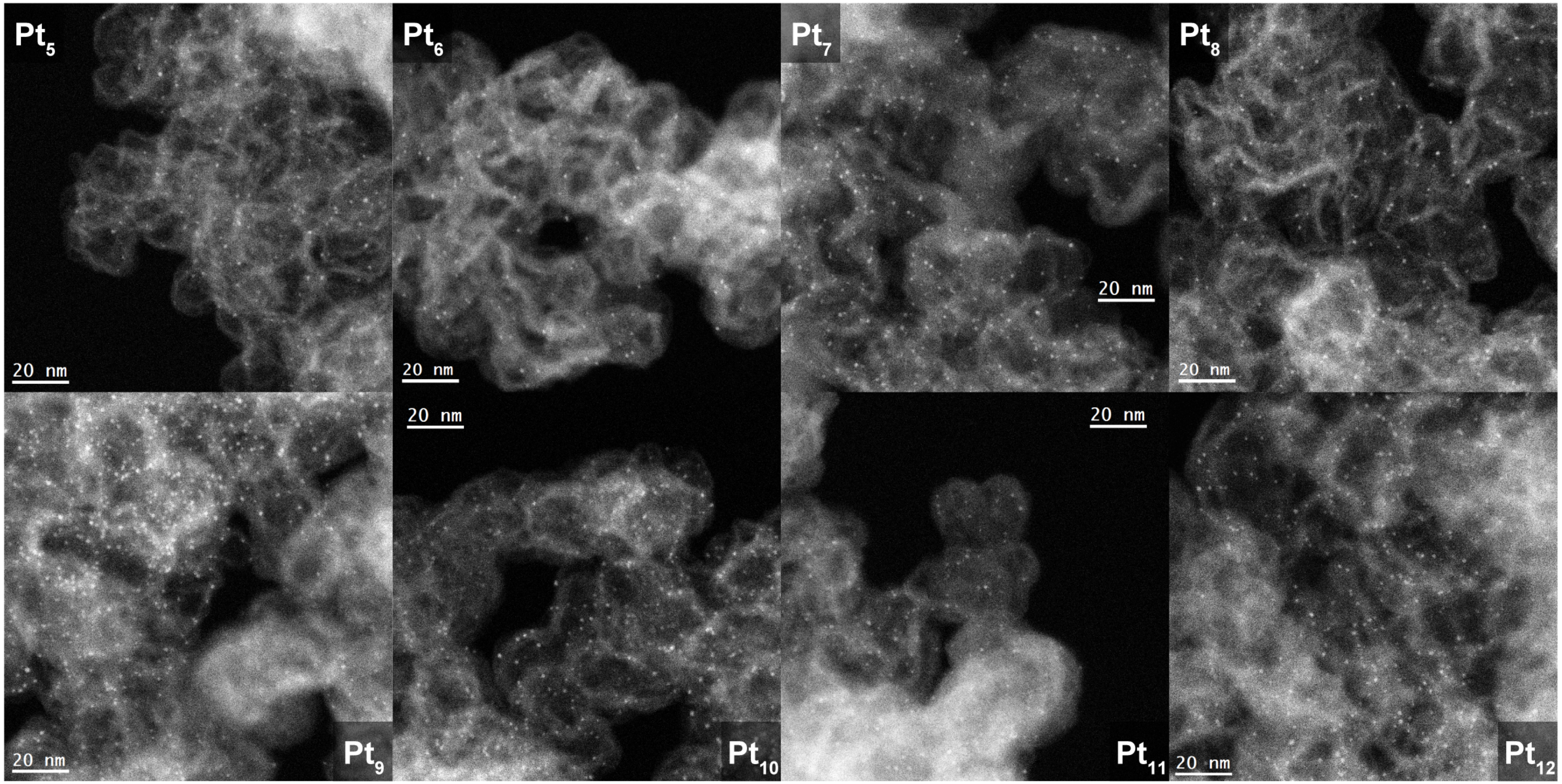

Supplementary Figure 6. Low-magnification dark-field STEM images of the calcined platinum subnanoparitcles from the platinum thiolate complexes with different atomicities supported on a carbon (Ketjenblack).

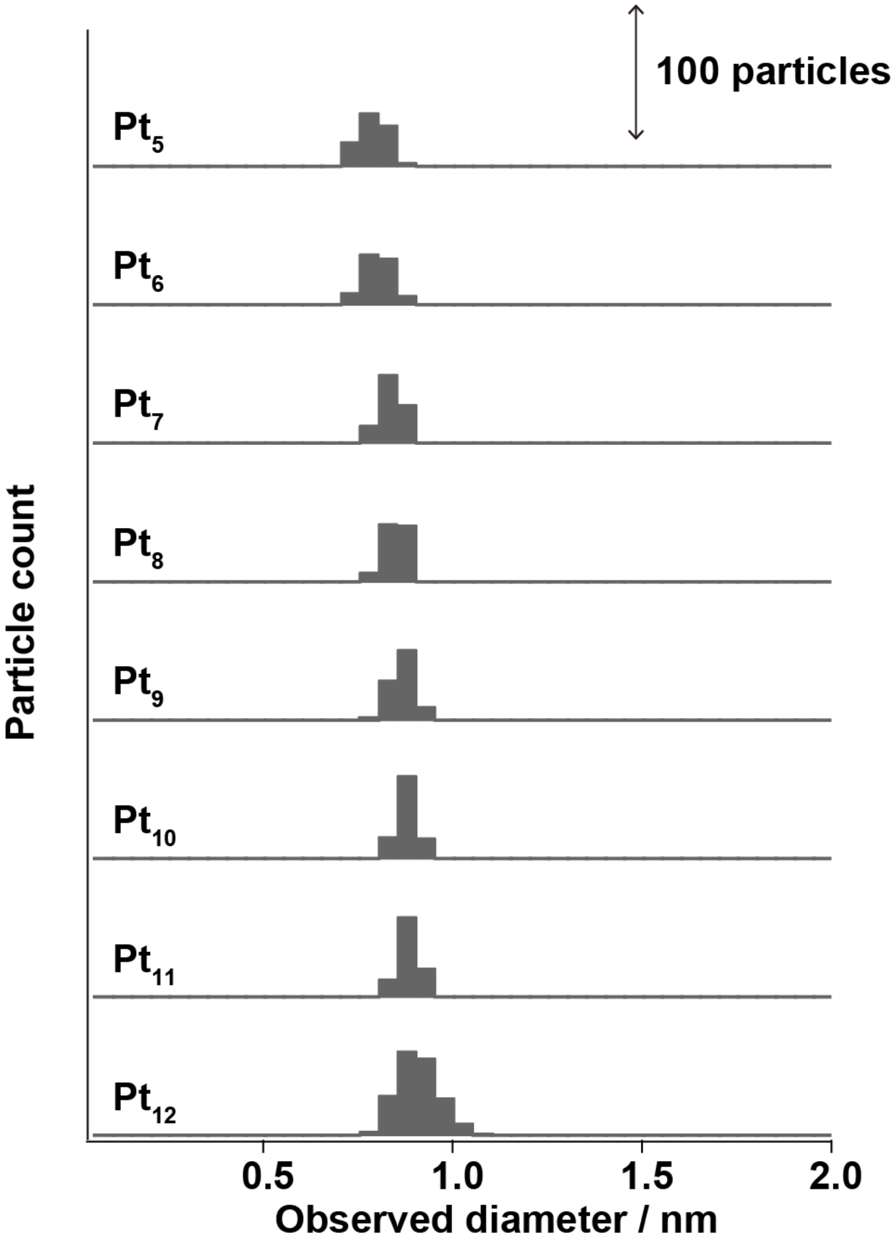

Supplementary Figure 7. Histograms of the particle size found in the HAADF-STEM images (Figure S6).

Before Calcination (thiolate complex)

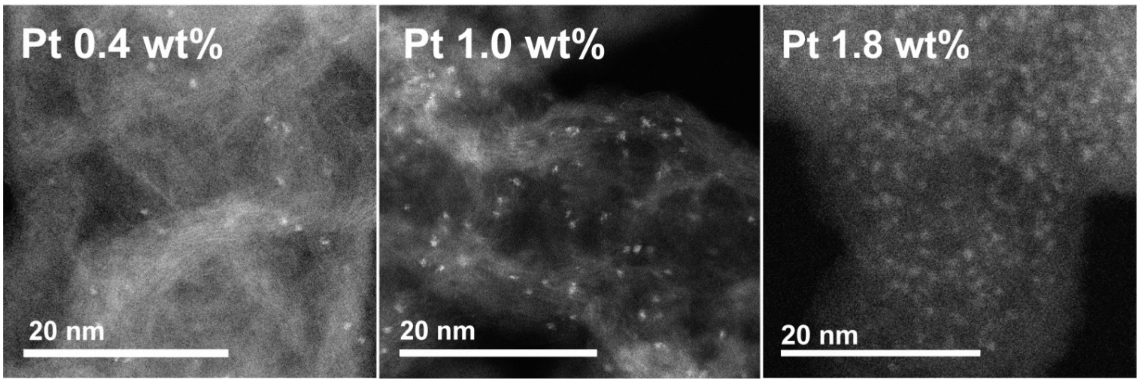

After Calcination (metal cluster)

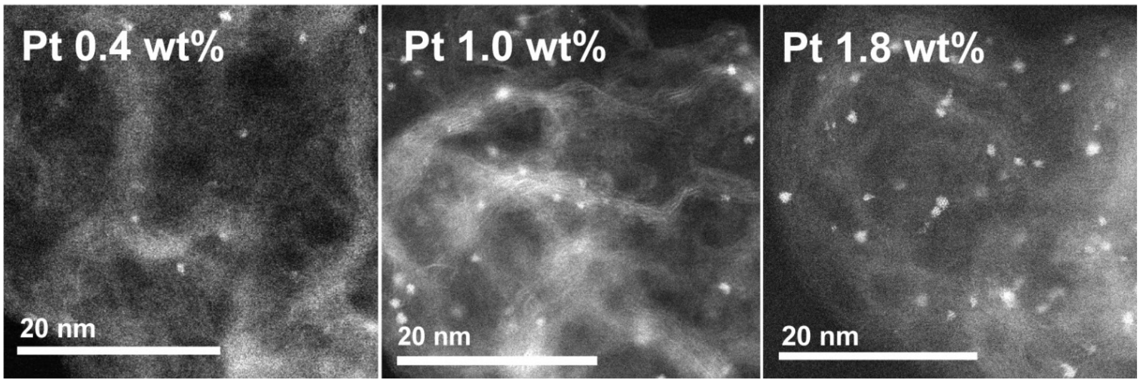

Supplementary Figure 8. Difference of the HAADF-STEM images of **[Pt($C_8H_{17}S$)$_2$]$_8$** (before) and **Pt$_8$** (after) at different weight percentage of platinum loading on Ketjenblack.

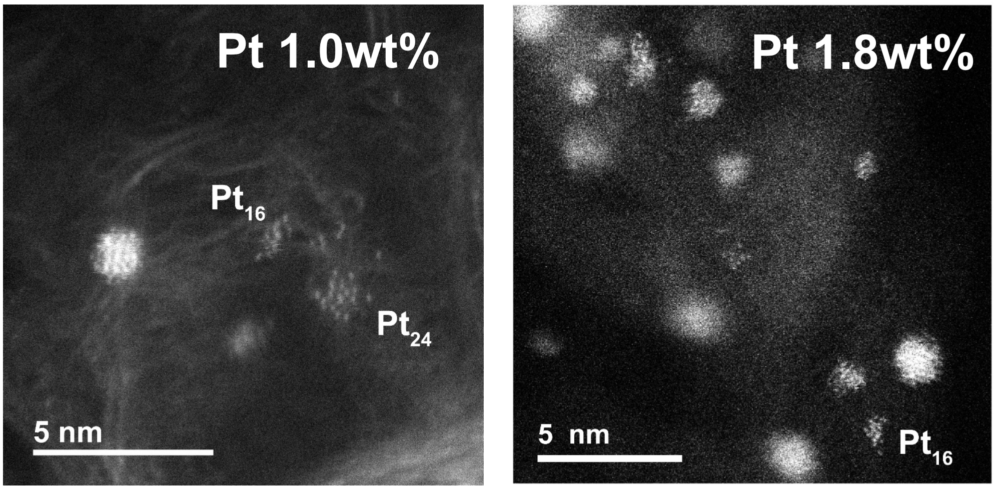

Supplementary Figure 9. Atomic scale HAADF-STEM images of **Pt_8** at higher weight percentages (Pt: 1.0 and 1.8 wt%) of platium loading on Ketjenblack. Most of the resulting clusters are aggregated producing larger clusters and nanoparticles.

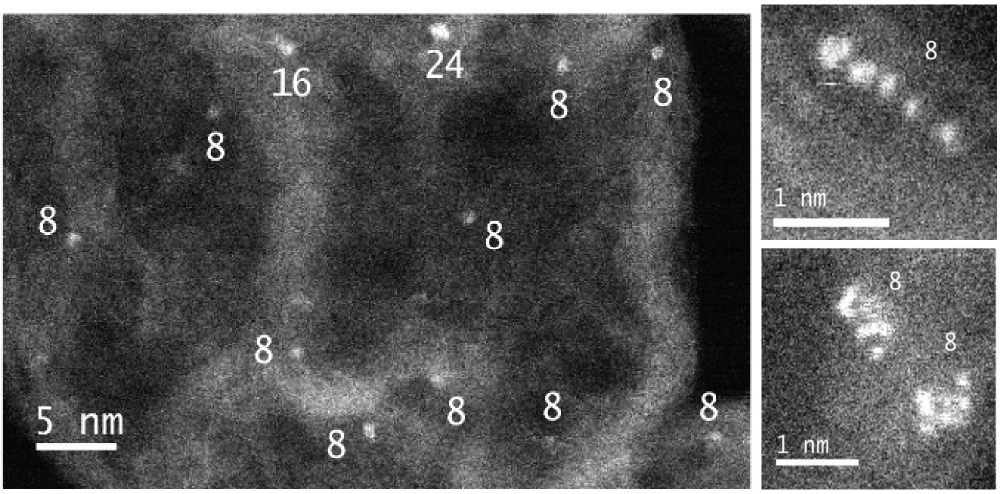

Supplementary Figure 10. A sub-atomic scale HAADF-STEM image of **Pt_8** on Ketjenblack (Pt: 0.4 wt%) and the magnifications of the respective clusters.

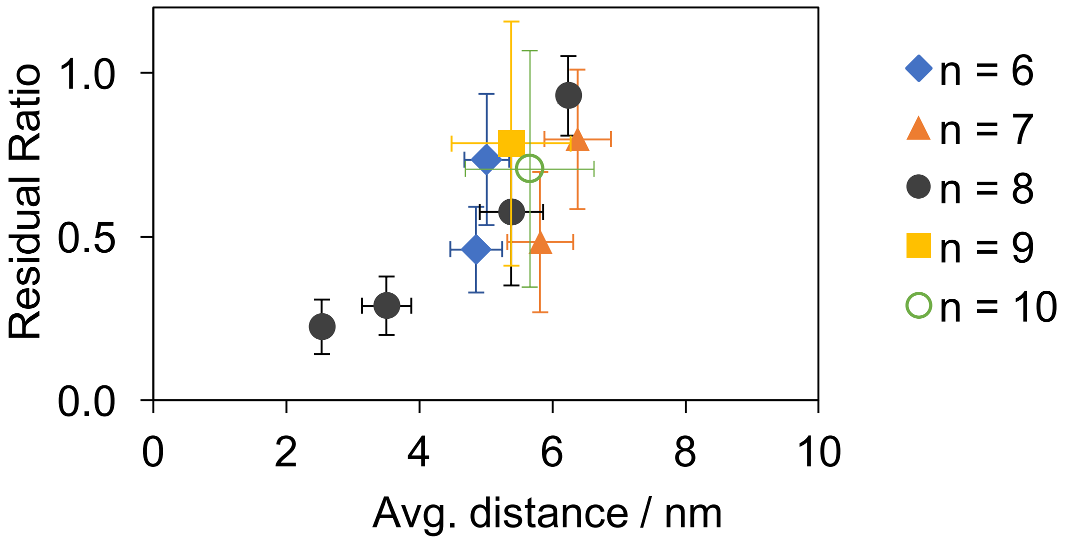

Supplementary Figure 11. Statistical analysis of the particle aggregation before and after the reductive calcination. Residual ratio (R) is defined as $R = N_{after}/N_{before}$ where N_{before} and N_{after} are the numbers of observed particles in a sampling area. Average distance was derived from the number of Pt-thiolate complexes (before the calcination) in the sampling area of the STEM image. Increase of the Pt loading (wt%) on the carbon support (Ketjenblack) decreases the distance. If the particles did not aggregate at all, R would be 1. This analysis suggest that the original particle could be preserved when the particle-to-particle distance was longer than ca. 7 nm.

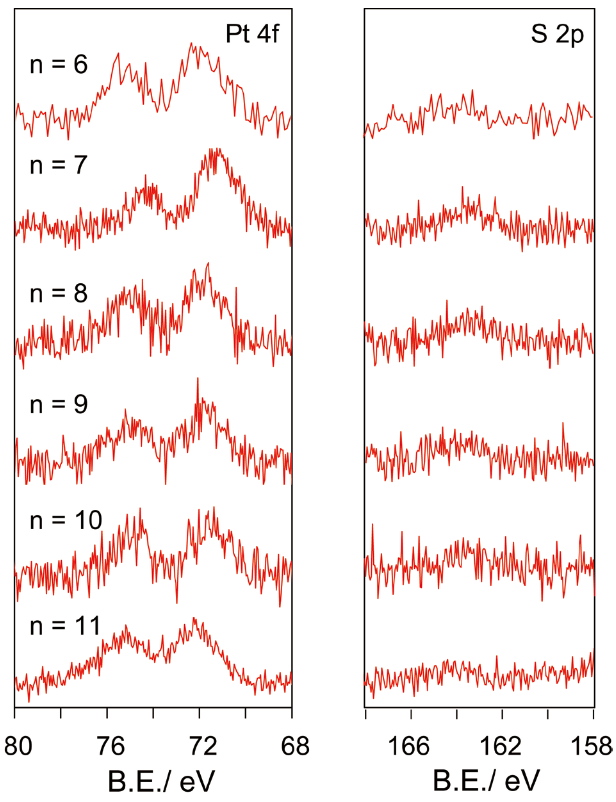

Supplementary Figure 12. X-ray photoelectron spectra (S 2*p* and Pt *4f*) of $\mathbf{Pt_x}$ (x = 6 - 11).

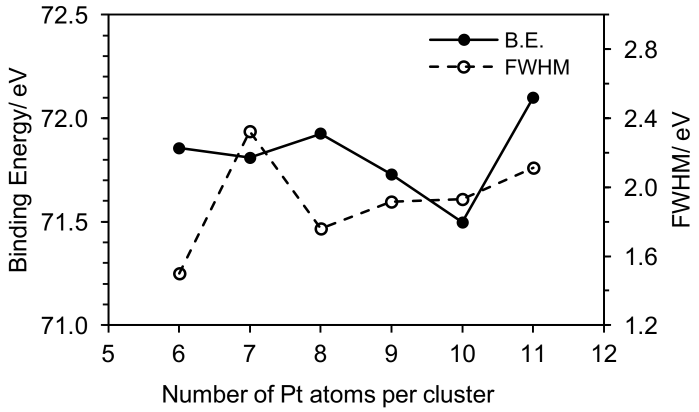

Supplementary Figure 13. Cluster-size dependences of binding energies and FWHM values from X-ray photoelectron spectra ($4f_{7/2}$).

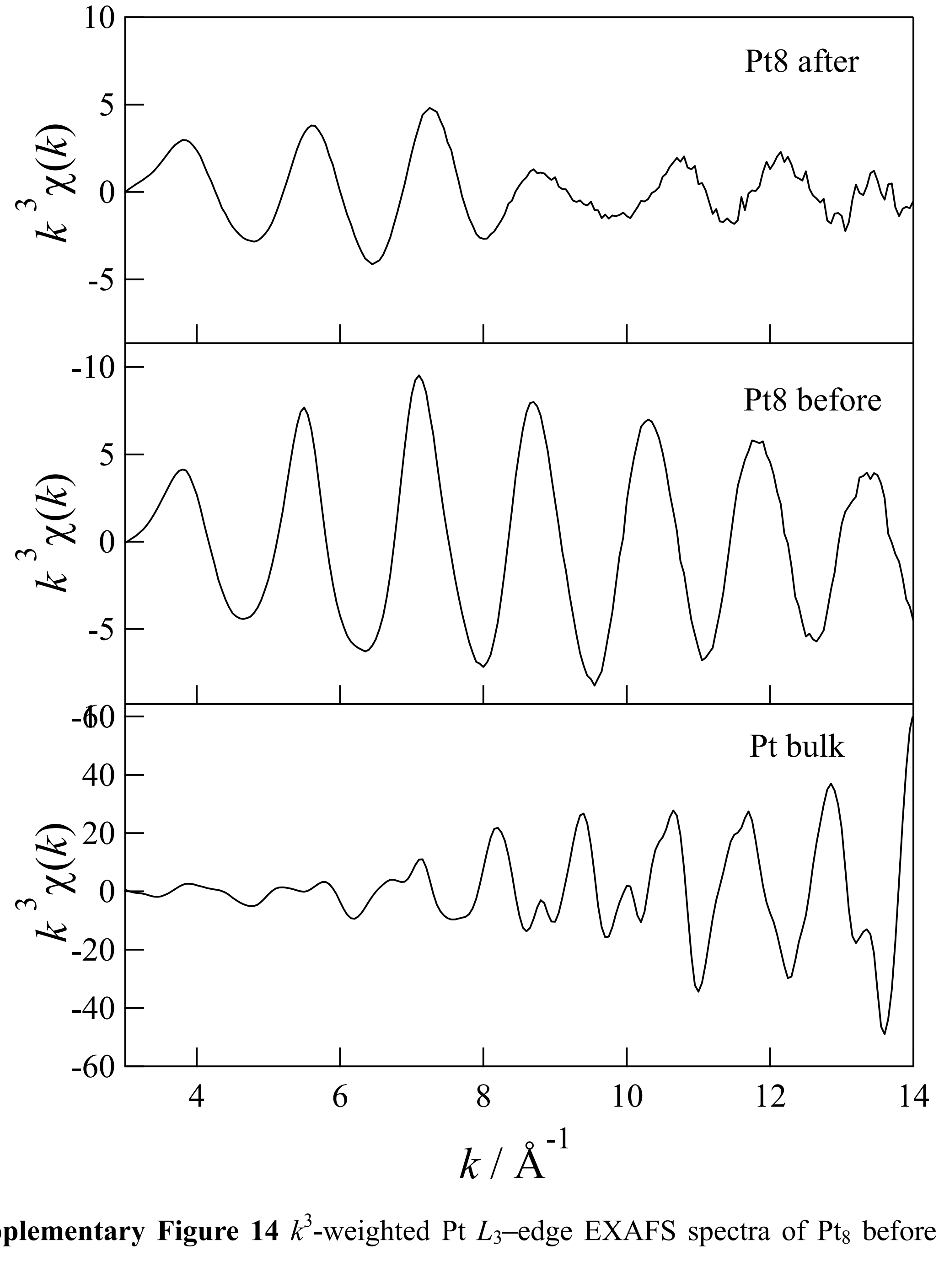

Supplementary Figure 14 k^3-weighted Pt L_3–edge EXAFS spectra of Pt_8 before and after calcination under H_2 stream with Pt foil as a reference.

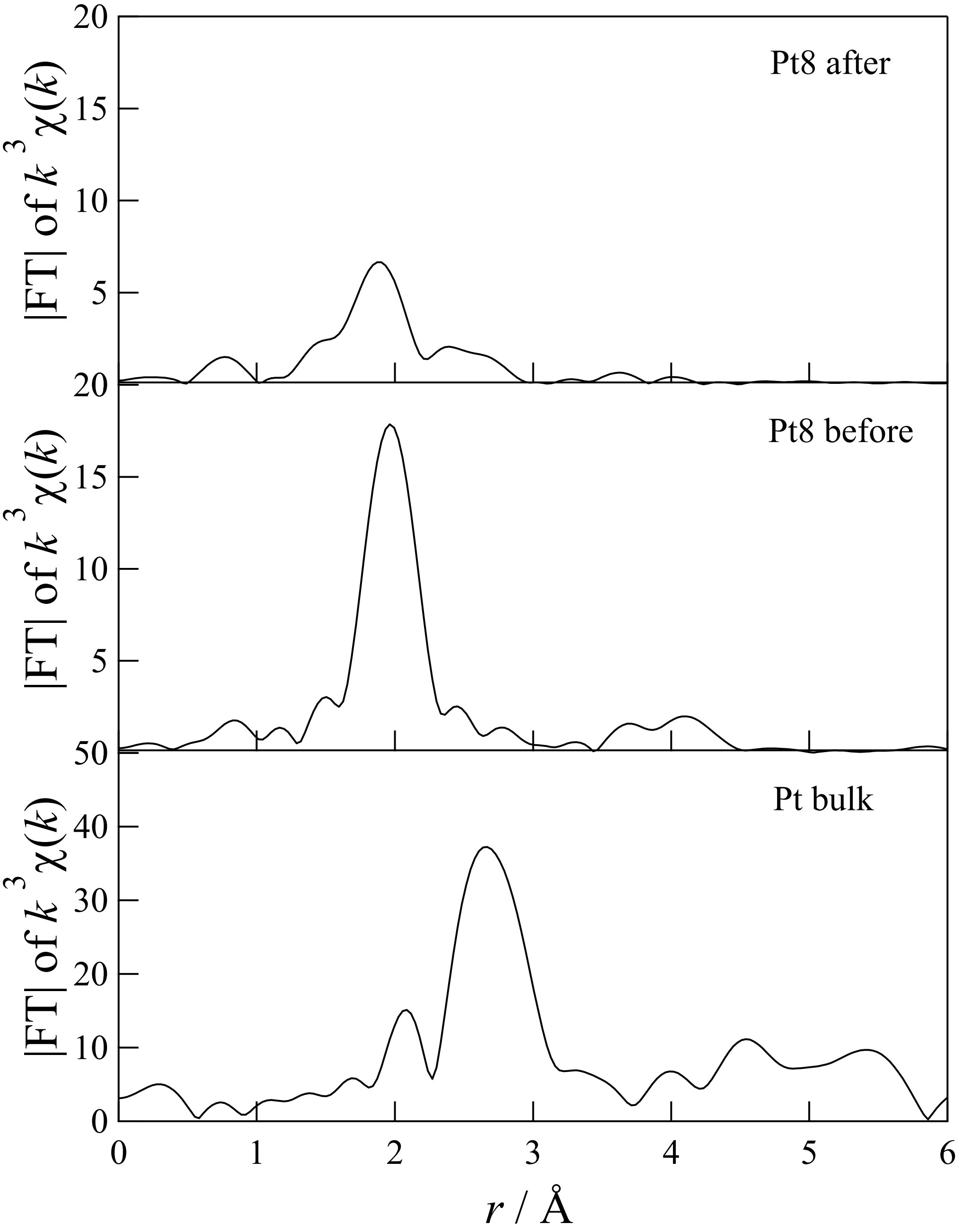

Supplementary Figure 15 Fourier-transformed k^3-weighted Pt L_3–edge EXAFS spectra of Pt_8 before and after calcination under H_2 stream with Pt foil as a reference.

*Fourier transform was limited where $\Delta k = 3.0 \sim 13.5$ Å^{-1} in EXAFS spectra.

Supplementary Table 2. Curve fitting results of the EXAFS of Pt_8 before and after calcination under H_2 stream.

sample	bond	N	r / Å	ΔE_0 / eV	σ^2/ 10^{-3}Å^2	R_f / %
before*	Pt-S	4.7 ± 0.7	2.32 ± 0.01	12.2 ± 2.0	2.70 ± 0.04	0.6
after**	Pt-C	6.6 ± 1.2	2.17 ± 0.01	21.8 ± 2.0	6.56 ± 0.36	0.9
	Pt-Pt	3.5 ± 2.1	2.71 ± 0.03	21.1 ± 6.4	11.24 ± 0.14	
Pt foil[a]	Pt-Pt	12	2.77	*n/a*	*n/a*	*n/a*

[a] Crystallographic data

Notations: N, coordination number; r, bond distance between absorber and backscatter atoms; σ^2, the Debye-Waller factor (DW); ΔE_0, the inner potential correction accounts for the difference in the inner potential between the sample and the reference; R_f (R-factor), a goodness of curve fit.

*Fourier transform and Fourier filtering region were limited where Δk = 3.0 ~ 13.5 Å^{-1} and Δr = 1.29 ~ 2.64 Å, respectively.

**Fourier transform and Fourier filtering region were limited where Δk = 3.0 ~ 13.5 Å^{-1} and Δr = 1.29 ~ 2.95 Å, respectively.

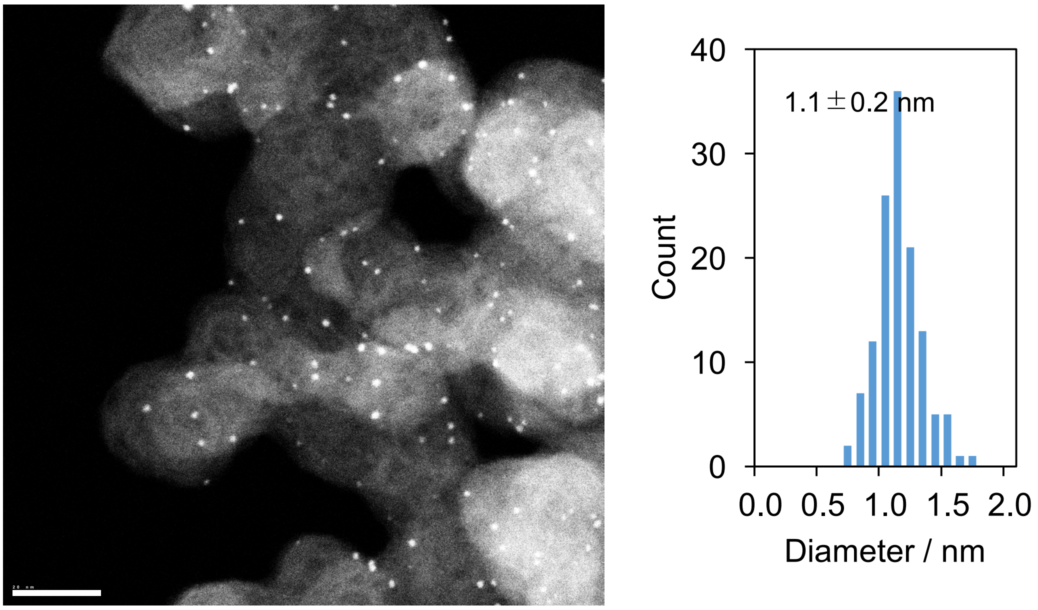

Supplementary Figure 16 A HAADF-STEM image (left) and the particle diameter histogram of larger platinum nanoparticles used as the reference catalyst for the styrene hydrogenation. This catalyst was synthesized from $[Pt(C_8H_{17}S)_2]_9$ supported on KB at 1.8 wt% (Pt atom weight percentage) as shown in the experimental section. Scale bar, 20 nm.

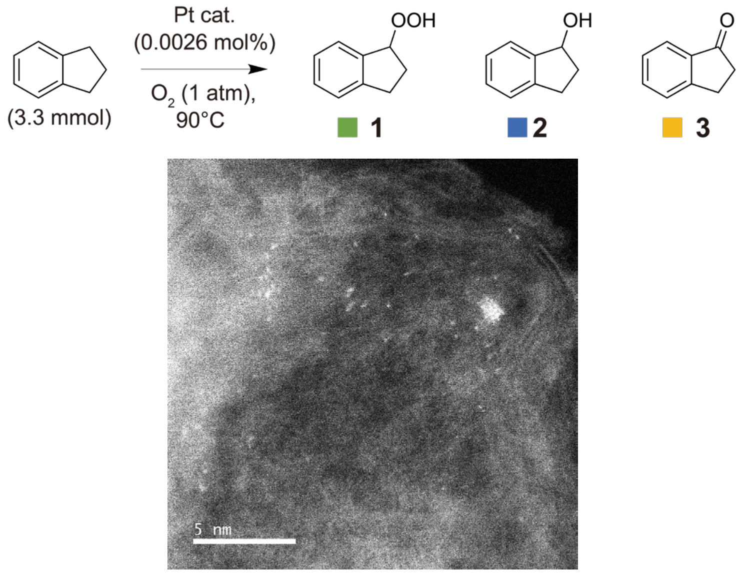

Supplementary Figure 17 A HAADF-STEM image of the Pt_8/KB catalyst at 4 hours of the aerobic catalytic oxidation of indane at 90°C. This image shows smaller clusters were decomposed to singular atoms. Scale bar, 5 nm.

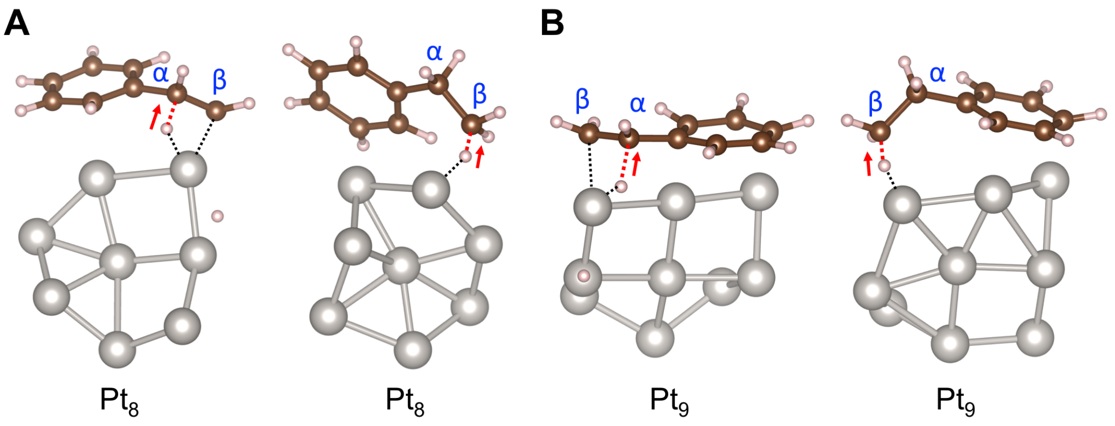

Supplementary Figure 18 Transition states of hydrogen insertions into α–carbon and β–carbon of styrene with (A) Pt_8 or (B) Pt_9. Density-functional-theory calculations were performed at the B3LYP/LanL2DZ and 6–31G (d, p) level of theory.

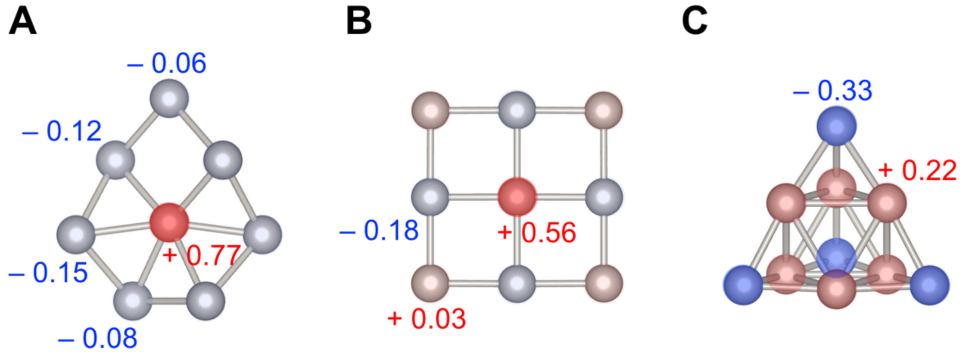

Supplementary Figure 19 Mulliken charge distributions of (A) Pt_8, (B) Pt_9, and (C) Pt_{10}. Density-functional-theory calculations were performed at the B3LYP/LanL2DZ level of theory.

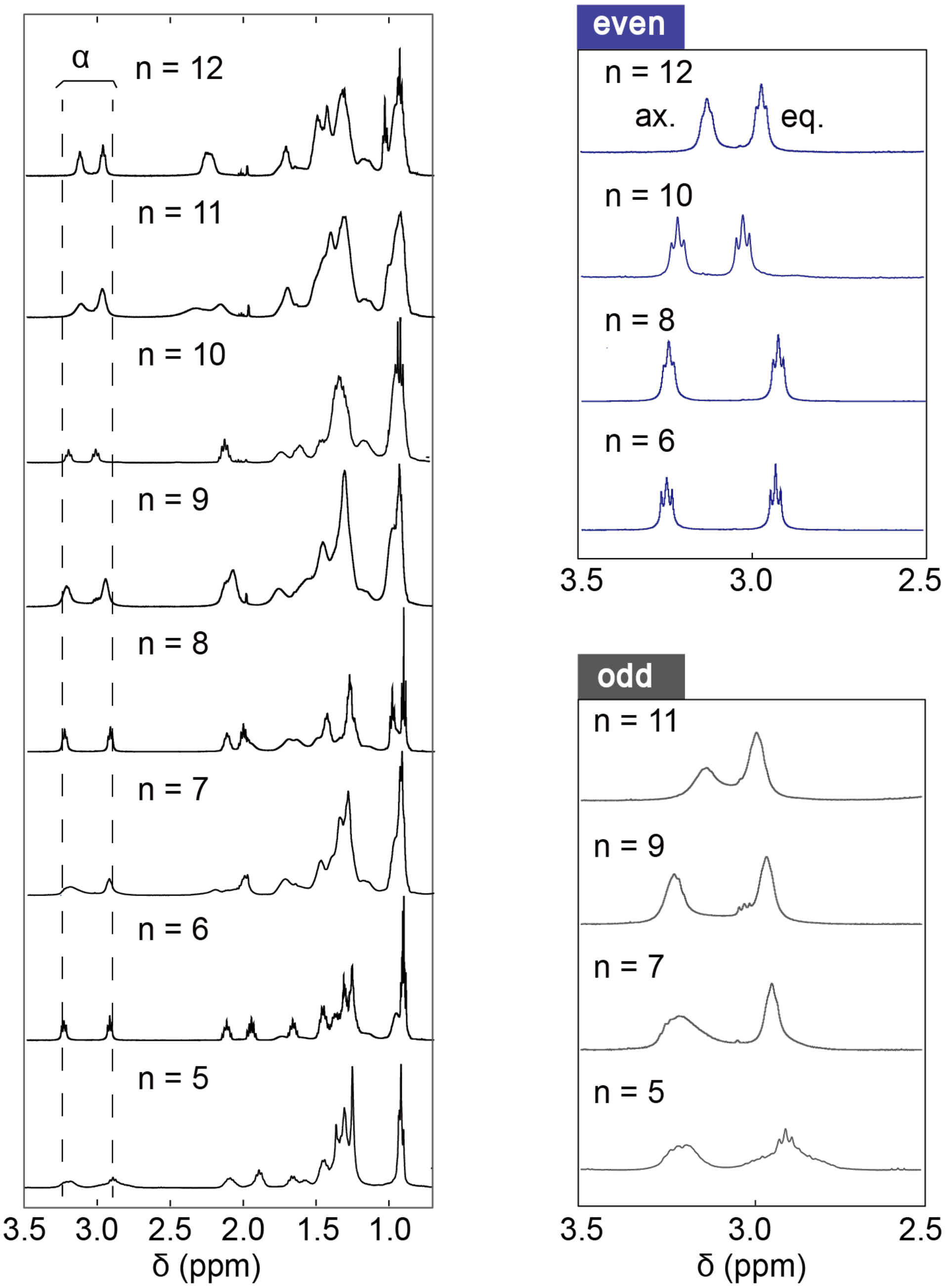

Supplementary Figure 20 ^{1}H NMR spectra of [Pt(μ-SC_8H_{17})$_2$]$_n$. Figures on the right side are the enlargements focusing on the α-proton region.

Supplementary Methods

STEM observation. Low magnification dark-field STEM images were obtained using a standard transmission electron microscope (JEOL, JEM-2100F) operated at a 200 kV acceleration. Atomic resolution high magnification STEM images were obtained using an aberration-corrected transmission electron microscope (JEOL, JEM-ARM200F) operated at 80 kV. The carbon-supported cluster samples were dispersed in a methanol solution, then cast on an elastic carbon film with a Cu mesh (Nisshin EM Co.) followed by vacuum drying at 40 °C overnight.

Calculations of platinum thiolate complexes. Structurer optimization of the platinum thiolate complexes were performed using the B3LYP functional as implemented in the Gaussian09 program. The effective core potential LANL2DZ basis set was chosen. The geometries of all the structures were fully optimized without any restriction. Validity of the optimized structure was checked by the absence of any imaginary frequency associated with the vibration analysis. The octanthiol ligands were replaced with methanethiol for all the complexes to reduce the calculation cost.

X-ray crystal structure determination. Single crystals of $\mathbf{[Pt(C_8H_{17}S)_2]_6}$ and $\mathbf{[Pt(C_8H_{17}S)_2]_8}$ suitable for X-ray diffraction were prepared by recrystallization in chloroform / methanol. Although we also tried to prepare the single crystals of other ring numbers, only n = 6 and 8 afforded the single crystals with enough quality.The diffraction data were collected by a Bruker APEXII ULTRA/CCD diffractometer or a Rigaku-XtaLAB P200 equipped with a Rigaku PILATUS3 R 200K area-detector system. The measurements were made using graphite monochromated Mo-$K\alpha$ radiation (λ = 0.71073 Å) at 77 K under a cold nitrogen stream. The structures were solved by direct methods using SHELXS-2013 and were refined using SHELXL-2014. Anisotropic refinement was applied to all the non-hydrogen atoms, and some of the

disordered atoms were isotopically refined. More detailed crystallographic data are listed in their cif files.

XAFS data acquisition and analysis. The Pt L_3-edge EXAFS was measured in the transmission mode at beamlines BL-9C and BL-12C of KEK-IMSS-PF. Synchrotron radiation from the storage ring (2.5 GeV, 400 mA) was monochromatized with Si(111) channel-cut crystals. The angle of the monochromator was calibrated using Pt foil. For suppressing the dynamic disorder, EXAFS data were recorded at cryo-cooled temperature (30K). Ionization chambers filled with 15% Ar-85% N_2 mixed gas and 100% Ar were used as detectors to monitor the incident (I_0) and transmitted X-rays (I), respectively.

EXAFS curve fitting results were obtained by using REX2000 package (Rigaku Co., Japan). The theoretical phase shifts and the amplitude functions for Pt-Pt, Pt-C, and Pt-S used in the fitting routine were calculated using the FEFF code[1] via Pt bulk, (π-C_5H_5)$PtMe_3$[2], [Pt($C_8H_{17}S$)$_2$]$_8$ crystallographic parameters, respectively.

Catalytic oxidation reaction of indan. Freshly distilled indan was added to a glass tube containing each platinum catalyst supported on a carbon. The molar amount of platinum was set to be 0.0026 mol% against indan. After the reaction for 6 hours at 90°C under an oxygen atmosphere (1 atm), the reaction mixture was analyzed by ^{1}H NMR displaying each product. The amounts of the products were determined by the integral values of each the product, which was referenced to anisole as the internal standard. A HAADF-STEM image of the used catalyst was sampled at 4 hours.

Chemical characterization. The matrix-assisted laser desorption ionization time-of-flight mass spectra (MALDI-TOF-MS) were obtained using a spectrometer (Bruker, Ultra flex: Positive ion mode). DCTB (trans-2-[3-(4-tert-butylphenyl)-2-methyl-2-propenylidene] malononitrile) was used as the matrix for the MALDI-TOF-mass measurements. The ^{1}H and ^{13}C nuclear magnetic resonance (NMR) measurements were recorded by an FT-NMR spectrometer (Bruker,

Avance III 400) operating at 400 MHz (^{1}H) or 100 MHz (^{13}C). The ^{1}H NMR chemical shifts were referenced to the signal of tetramethoxysilane (TMS : 0 ppm) as the internal standard. The ^{13}C NMR chemical shifts were referenced to the solvent peak ($CDCl_3$: 77.0 ppm). The XPS spectra were obtained using a spectrometer (Ulvac-Phi, ESCA1700R) with Mg Kα radiation.

Characterization data

[Pt(C_8H_{17}S)$_2$]$_5$. ^{1}H NMR (400MHz, benzene-d_6, 303K, δ in ppm) 3.21 (10H, br, S-CH_2-), 2.90 (10H, br, S-CH_2-), 2.09 (10H, br, S-CH_2-CH_2-), 1.89 (10H, br, S-CH_2-CH_2-), 1.67-1.58 (100H, m, -CH_2-CH_2-CH_2-), 0.92 (30H, m, -CH_3). ^{13}C NMR (100 MHz, bezene-d_6, δ in ppm) 32.77, 30.61, 30.26, 30.12, 29.70, 23.49, 14.75. DOSY NMR (dichlorometahne-d_2, 303 K) $D = 6.0 \times 10^{10}$ m^2 s^{-1}. MALDI-TOF-MS. *m/z* Calcd for $Pt_5C_{80}H_{170}S_{10}$ (M^+): 2428.88. Found 2428.52. Anal. Calcd for $Pt_5C_{80}H_{170}S_{10}$: C, 39.57; H, 7.06; S, 13.20. Found : C, 39.90; H, 6.78; S, 13.34. Yield 6.8 mg.

[Pt(C_8H_{17}S)$_2$]$_6$. ^{1}H NMR (400MHz, benzene-d_6, 303K, δ in ppm) 3.23 (12H, t, J = 7.6 Hz, S-CH_2-), 2.92 (12H, t, J = 7.4 Hz, S-CH_2-), 2.12 (12H, tt, J = 7.4, 7,4 Hz, S-CH_2-CH_2-), 1.96 (12H, tt, J = 7.4, 7,4 Hz, S-CH_2-CH_2-), 1.68 (12H, tt, J = 7.4, 7,4 Hz -CH_2-CH_2-CH_2-), 1.5-1.2 (108H, m, -CH_2-CH_2-CH_2-), 0.92 (36H, m). ^{13}C NMR (100 MHz, bezene-d_6, δ in ppm) 37.11, 33.30, 32.83, 32.70, 31.15, 30.75, 30.35, 30.18, 30.10, 30.03, 29.66, 23.58, 23.51, 14.76, 14.73. DOSY NMR (dichlorometahne-d_2, 303 K) $D = 6.1 \times 10^{10}$ m^2 s^{-1}. MALDI-TOF-MS. *m/z* Calcd for $Pt_6C_{96}H_{204}S_{12}$ (M^+): 2914.06. Found 2913.97. Anal. Calcd for $Pt_6C_{96}H_{204}S_{12}$: C, 39.57; H, 7.06; S, 13.20. Found : C, 39.46; H, 6.97; S, 13.29. Yield 12.4 mg.

[Pt(C_8H_{17}S)$_2$]$_7$. ^{1}H NMR (400MHz, benzene-d_6, 303K, δ in ppm) 3.22 (14H, br, S-CH_2-), 2.94 (14H, br, S-CH_2-), 2.19 (14H, br, S-CH_2-CH_2-), 2.00 (14H, br,

S-CH_2-CH_2-), 1.7-1.2 (140H, m, br, -CH_2-CH_2-CH_2-), 0.94 (42H, br). ^{13}C NMR (100 MHz, bezene-d_6, δ in ppm) 32.74, 30.61, 30.22, 30.22, 23.51, 14.77. $D = 4.4 \times 10^{10}$ m^2 s^{-1}. MALDI-TOF-MS. *m/z* Calcd for $Pt_7C_{112}H_{238}S_{14}$ (M^+): 3400.23. Found 3400.42. Anal. Calcd for $Pt_7C_{112}H_{238}S_{14}$: C, 39.57; H, 7.06; S, 13.20. Found : C, 39.62; H, 7.01; S, 12.97. Yield 9.7 mg.

[Pt($C_8H_{17}S$)$_2$]$_8$. ^{1}H NMR (400MHz, benzene-d_6, 303K, δ in ppm) 3.24 (16H, t, *J* = 7.4 Hz, S-CH_2-), 2.94 (16H, t, *J* = 7.4 Hz, S-CH_2-), 2.14 (16H, tt, *J* = 7.4, 7,4 Hz, S-CH_2-CH_2-), 2.03 (16H, tt, *J* = 7.4, 7,4 Hz, S-CH_2-CH_2-), 1.68 (16H, tt, *J* = 7.4, 7,4 Hz), 1.8-1.2 (160H, m, br, -CH_2-CH_2-CH_2-), 1.0-0.9 (48H, m). ^{13}C NMR (100 MHz, bezene-d_6, δ in ppm) 32.72, 30.60, 30.18, 30.18, 29.79, 23.52, 14.77. $D = 5.1 \times 10^{10}$ m^2 s^{-1}. MALDI-TOF-MS. *m/z* Calcd for $Pt_8C_{128}H_{272}S_{16}$ (M^+): 3885.40. Found 3885.76. Anal. Calcd for $Pt_8C_{128}H_{272}S_{16}$: C, 39.57; H, 7.06; S, 13.20. Found : C, 39.49 ; H, 7.03 ; S, 12.82. Yield 16.9 mg.

[Pt($C_8H_{17}S$)$_2$]$_9$. ^{1}H NMR (400MHz, benzene-d_6, 303K, δ in ppm) 3.22 (18H, br, S-CH_2-), 2.96 (18H, br, S-CH_2-), 2.1 (36H, br, S-CH_2-CH_2-), 1.9-1.2 (180H, m, br, -CH_2-CH_2-CH_2-), 0.93 (54H, br). ^{13}C NMR (100 MHz, bezene-d_6, δ in ppm) 32.94, 31.28, 30.80, 30.42, 30.16, 23.72, 14.96. $D = 4.7 \times 10^{10}$ m^2 s^{-1}. MALDI-TOF-MS. *m/z* Calcd for $Pt_9C_{144}H_{306}S_{18}$ (M^+): 4371.58. Found 4372.18. Anal. Calcd for $Pt_9C_{144}H_{306}S_{18}$: C, 39.57; H, 7.06; S, 13.20. Found : C, 39.63 ; H, 7.06 ; S, 13.08. Yield 13.0 mg.

[Pt($C_8H_{17}S$)$_2$]$_{10}$. ^{1}H NMR (400MHz, benzene-d_6, 303K, δ in ppm) 3.21 (20H, t, *J* = 7.4 Hz, S-CH_2-), 3.02 (20H, t, *J* = 7.4 Hz, S-CH_2-), 2.14 (40H, t, *J* = 7.4, 7,4 Hz, S-CH_2-CH_2-), 1.6-1.2 (200H, m, -CH_2-CH_2-CH_2-), 0.93 (60H, m). ^{13}C NMR (100 MHz, bezene-d_6, δ in ppm) 34.45, 32.83, 32.72, 30.50, 30.20, 30.17, 29.87, 23.51, 14.76. $D = 5.2 \times 10^{10}$ m^2 s^{-1}. MALDI-TOF-MS. *m/z* Calcd for $Pt_{10}C_{160}H_{340}S_{20}$ (M^+): 4856.75.

Found 4857.64. Anal. Calcd for $Pt_{10}C_{160}H_{340}S_{20}$: C, 39.57; H, 7.06; S, 13.20. Found : C, 39.58 ; H, 7.07 ; S, 12.90. Yield 7.4 mg.

$[Pt(C_8H_{17}S)_2]_{11}$. ^{1}H NMR (400MHz, benzene-d_6, 303K, δ in ppm) 3.2-2.9 (44H, m, br, S-CH_2-), 2.4-2.1 (44H, m, br, S-CH$_2$-CH_2-), 1.8-1.2 (220H, br, -CH$_2$-CH$_2$-CH_2-), 1.1-0.9 (66H, br). ^{13}C NMR (100 MHz, bezene-d_6, δ in ppm) 34.67, 32.73, 30.59, 30.21, 23.51, 23.08, 14.76, 14.62. $D = 5.4 \times 10^{10}$ m^2 s^{-1}. MALDI-TOF-MS. *m/z* Calcd for $Pt_{11}C_{176}H_{374}S_{22}$ (M^+): 5342.92. Found 5344.06. Anal. Calcd for $Pt_{11}C_{176}H_{374}S_{22}$: C, 39.57; H, 7.06; S, 13.20. Found : C, 39.59 ; H, 7.17 ; S, 13.11. Yield 7.8 mg.

$[Pt(C_8H_{17}S)_2]_{12}$. ^{1}H NMR (400MHz, benzene-d_6, 303K, δ in ppm) 3.13 (24H, t, J = 7.4 Hz, S-CH_2-), 2.97 (24H, t, J = 7.4 Hz, S-CH_2-), 2.25 (48H, br, S-CH$_2$-CH_2-), 1.7-1.2 (240H, br, m, -CH$_2$-CH$_2$-CH_2-), 1.1-0.8 (72H, m). ^{13}C NMR (100 MHz, bezene-d_6, δ in ppm) 34.94, 33.03, 32.85, 30.63, 30.32, 23.84, 23.65, 23.20, 14.89, 14.73. $D = 4.5 \times 10^{10}$ m^2 s^{-1}. MALDI-TOF-MS. *m/z* Calcd for $Pt_{12}C_{192}H_{408}S_{24}$ (M^+): 5828.10. Found 5829.45. Anal. Calcd for $Pt_{12}C_{192}H_{408}S_{24}$: C, 39.57; H, 7.06; S, 13.20. Found : C, 39.48 ; H, 7.14 ; S, 13.12. Yield 4.8 mg.

Supplementary References

1. Rehr, J. J. & Albers, R. C. Scattering-matrix formulation of curved-wave multiple-scattering theory: Application to x-ray-absorption fine structure. *Phys. Rev. B* **41,** 8139–8149 (1990).
2. Adamson, G. W., Bart, J. C. J. & Daly, J. J. Crystal and molecular structure of cyclopentadienyl(trimethyl)platinum(IV), (π-C_5H_5)$PtMe_3$. *J. Chem. Soc., A***,** 2616–2619 (1971).
3. Yamamoto, K. *et al.* Size-specific catalytic activity of platinum clusters enhances oxygen reduction reactions. *Nature Chem.* **1,** 397–402 (2009).
4. Chaves, A. S. *et al*. The Role of Charge States in the Atomic Structure of Cu_n and Pt_n (n = 2–14 atoms) Clusters: A DFT Investigation. *J. Phys. Chem.* A 118, 10813–10821 (2014).
5. Fung, V. & Jiang, D.–E. Exploring Structural Diversity and Fluxionality of Pt_n (n = 10–13) Clusters from First–Principles. *J. Phys. Chem. C* **121**, 10796–10802 (2017).
